# Nanoplasmonic Sensor Detects Preferential Binding of IRSp53 to Negative Membrane Curvature

**DOI:** 10.3389/fchem.2019.00001

**Published:** 2019-02-04

**Authors:** Gustav Emilsson, Evelyn Röder, Bita Malekian, Kunli Xiong, John Manzi, Feng-Ching Tsai, Nam-Joon Cho, Marta Bally, Andreas Dahlin

**Affiliations:** ^1^Pharmaceutical Sciences, AstraZeneca R&D, Mölndal, Sweden; ^2^Department of Chemistry and Chemical Engineering, Chalmers University of Technology, Göteborg, Sweden; ^3^Laboratoire Physico Chimie Curie, Institut Curie, PSL Research University, CNRS UMR168, and Sorbonne Université, Paris, France; ^4^School of Materials Science and Engineering, Nanyang Technological University, Singapore, Singapore; ^5^Department of Clinical Microbiology & Wallenberg Centre for Molecular Medicine, Umeå University, Umeå, Sweden

**Keywords:** membranes, curvature, IRSp53, insulin receptor tyrosine kinase substrate p53, plasmons, sensors

## Abstract

Biosensors based on plasmonic nanostructures are widely used in various applications and benefit from numerous operational advantages. One type of application where nanostructured sensors provide unique value in comparison with, for instance, conventional surface plasmon resonance, is investigations of the influence of nanoscale geometry on biomolecular binding events. In this study, we show that plasmonic “nanowells” conformally coated with a continuous lipid bilayer can be used to detect the preferential binding of the insulin receptor tyrosine kinase substrate protein (IRSp53) I-BAR domain to regions of negative surface curvature, i.e., the interior of the nanowells. Two different sensor architectures with and without an additional niobium oxide layer are compared for this purpose. In both cases, curvature preferential binding of IRSp53 (at around 0.025 nm^−1^ and higher) can be detected qualitatively. The high refractive index niobium oxide influences the near field distribution and makes the signature for bilayer formation less clear, but the contrast for accumulation at regions of negative curvature is slightly higher. This work shows the first example of analyzing preferential binding of an average-sized and biologically important protein to negative membrane curvature in a label-free manner and in real-time, illustrating a unique application for nanoplasmonic sensors.

## Introduction

Biosensors based on plasmonic nanostructures have been a subject of intense research for 20 years (Jackman et al., 2017; Lopez et al., 2017; Zhang et al., 2017). Several transducer mechanisms exist (Xin et al., 2018), but the most straightforward detection principle is to immobilize a receptor on the sensor surface and detect analyte binding thanks to the local refractive index increase. This enables label-free and real-time biomolecular interaction analysis just like with the conventional surface plasmon resonance (SPR) technique. One key advantage of using nanostructured plasmonic sensors is that single molecule resolution is sometimes possible (Taylor and Zijlstra, 2017). Although technically impressive, actual detection of a single molecule from a complex sample is still not feasible due to complications such as mass transport limitations and non-specific adsorption. In fact, in most practical situations, the resolution in surface coverage (molecules per area) becomes more important as it determines the signal after equilibrium establishment with a given bulk concentration (Dahlin, 2012). Indeed, SPR remains the golden standard for biomolecular interaction analysis (Rich and Myszka, 2011) because of its high resolution in surface coverage (~0.01 ng/cm^2^). In addition, SPR is based on a single-material planar interface which simplifies functionalization and quantification.

In some recent studies, we have suggested that one unique type of application for plasmonic nanostructures is to study the effects of lipid membrane curvature on biomolecular interactions (Junesch et al., 2015; Ferhan et al., 2018). By depositing a conformal silica coating (Im et al., 2010), a lipid membrane which follows the surface curvature can be prepared by vesicle fusion (Jonsson et al., 2007). Using plasmonic nanowells (Junesch et al., 2012; Malekian et al., 2017), we have shown that proper spectral analysis can detect whether binding occurs preferentially to negative curvature, i.e., at the membrane invaginations formed inside the nanowells (Junesch et al., 2015; Ferhan et al., 2018). Negative membrane curvature is particularly difficult to study with vesicles and tubules since one has to access the interior volume (Prévost et al., 2015), in contrast to positive curvature for which binding to the outer vesicle surface can be analyzed. However, we have not yet shown that our nanoplasmonic sensor concept is applicable to proteins binding to membranes with negative curvature. Furthermore, the optimal design of the plasmonic nanostructure for improving the optical signals indicating bilayer formation and curvature-preferential binding has not been investigated.

**Scheme 1:**
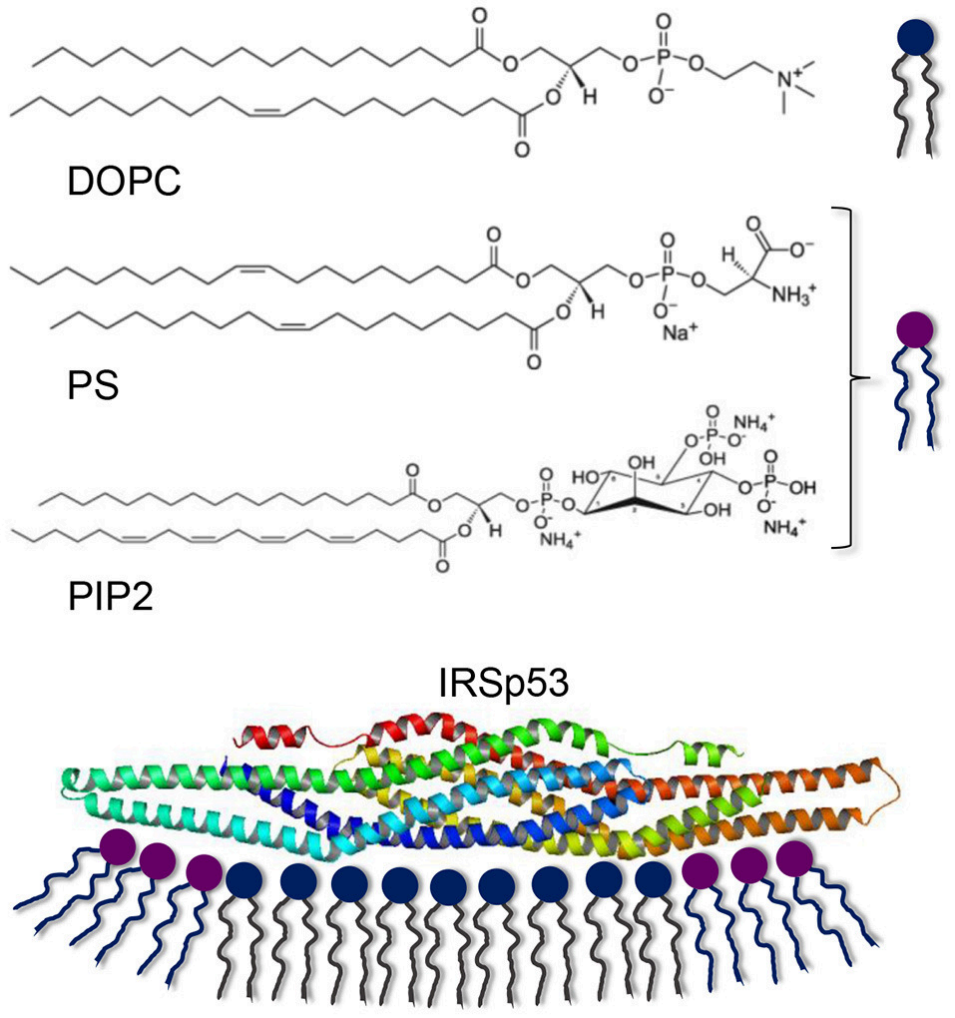
Lipids used for bilayer formation and the IRSp53 protein interacting with a membrane. Note that PS and PIP2 are net negatively charged (Na^+^ and ${\text{NH}}_{4}^{+}$ are counterions from salt formation). The electrostatic interaction with lipid bilayers is believed to occur with negatively charged lipids at the outer rim of the elongated protein, promoting a concave membrane shape.

In this work we evaluate the curvature-dependent interaction of the I-BAR domain of the IRSp53 protein with membranes containing negatively charged lipids (Mattila et al., 2007). IRSp53 is an eukaryotic inverse-BAR domain containing protein (Scheme 1) and thus believed to be involved in processes associated with cell membrane deformation (Frost et al., 2009; Saarikangas et al., 2009). Previous work has shown preferential binding and clustering at negative membrane curvature by fluorescent imaging of labeled proteins inside nanotubules formed from giant unilamellar vesicles clamped by micropipettes (Prévost et al., 2015). The advantages of our nanoplasmonic sensor are that it provides a much simpler methodology and operates with label-free readout. Experimental results are complemented with numerical simulations. We also discuss the ideal nanostructure design as well as the strengths and limitations of this concept for future investigations of proteins interacting with membranes exhibiting negative curvature.

## Experimental

All lipids were purchased from Avanti Polar Lipids. For binding of NeutrAvidin, 5% 1,2-dioleoyl-sn-glycero-3-phosphoethanolamine-N-(cap biotinyl) was introduced. Vesicles were prepared by drying the lipids onto the interior of a flask for 30 min, followed by hydration in buffer and extrusion 11 times through 30 nm pores (1 bar). The I-BAR domain of IRSp53 was produced as described previously (Saarikangas et al., 2009). Standard chemicals for buffer preparation were from Sigma. NeutrAvidin was from ThermoFisher.

Nanowells were prepared as described previously (Junesch et al., 2012, 2015; Malekian et al., 2017; Ferhan et al., 2018) using 107 nm polystyrene colloids on Nb_2_O_5_ and 158 nm on SiO_2_ (Microparticles). Nanowells in SiO_2_ were prepared on fused silica to enable direct etching of the solid support (Malekian et al., 2017). Nanowells in Nb_2_O_5_ were prepared on borosilicate glass (which cannot be easily etched) onto which Nb_2_O_5_ was first deposited by reactive sputter coating with O_2_ and Ar (Junesch et al., 2012) (Nordiko). A 20 nm thick SiO_2_ layer was deposited by plasma enhanced chemical vapor deposition (Surface Technology Systems). Recipes aiming for stochiometric SiO_2_ or Si_3_N_4_ were used.

For bilayer formation with negative lipids, a 20 mM citric acid buffer was used with 150 mM KCl at a pH of 4.8. IRSp53 binding was performed in a buffer with 20 mM tris(hydroxymethyl) aminomethane and 150 mM NaCl with pH adjusted to 7.4 unless stated otherwise. The pH values were adjusted with concentrated HCl and NaOH.

The setup for extinction spectroscopy with high resolution and tracking of multiple resonance features has been described previously (Junesch et al., 2015; Ferhan et al., 2018). Extinction is presented using the natural logarithm of the ratio between reference and measured intensities.

## Results and Discussion

**Figure 1:**
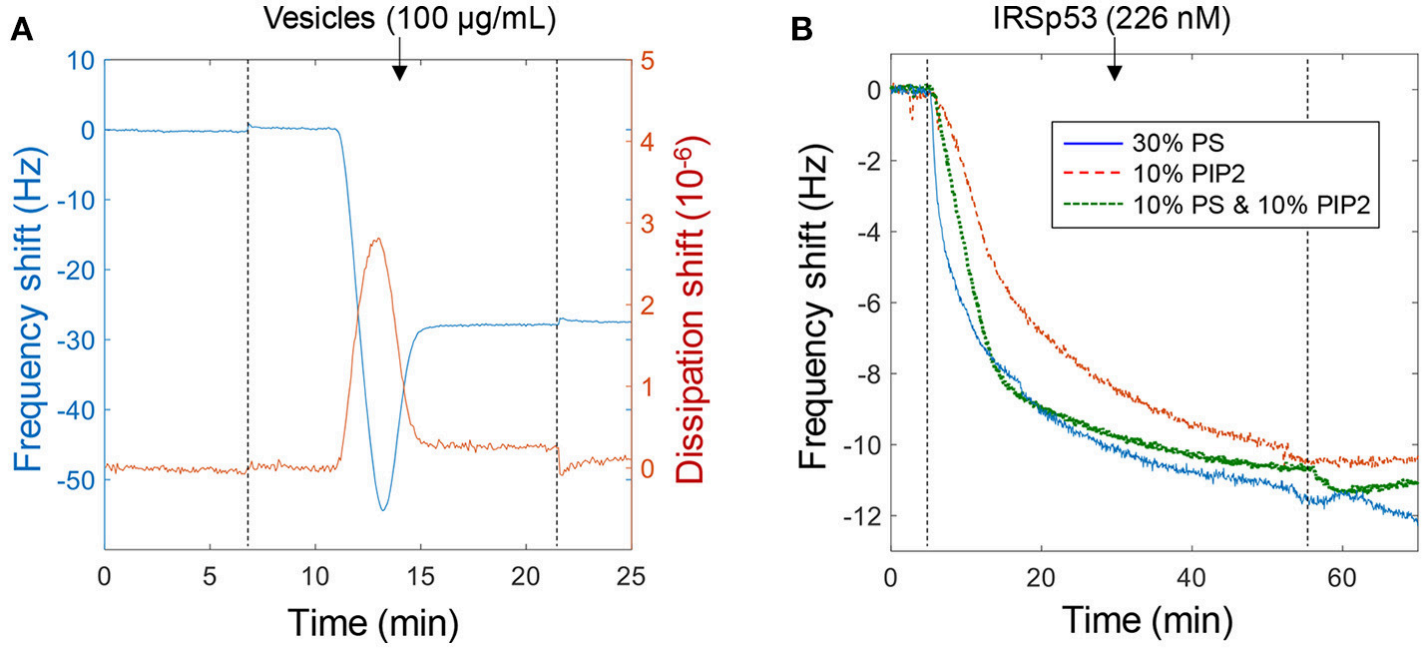
Using QCMD to verify the biomolecular interaction. **(A)** Example of bilayer formation on planar SiO_2_ with lipids containing 10% PS, 10% PIP2, and 80% DOPC. **(B)** Binding of IRSp53 at a relatively low concentration to different lipid compositions with negative charge (No binding was observed to pure DOPC bilayers). Note that for these curves the signal approaches ~12 Hz simply due to depletion of molecules in the liquid volume. Binding is irreversible as illustrated upon rinsing (dotted lines).

We first used the quartz crystal microbalance with dissipation monitoring (QCMD) technique to characterize the interaction between IRSp53 (the I-BAR domain) and solid supported lipid bilayer membranes. The IRSp53 protein is believed to interact with negatively charged lipids and induce domain formation (Scheme 1) (Saarikangas et al., 2009). We used 1,2-dioleoyl-*sn*-glycero-3-phosphocholine (DOPC) as the main (zwitterionic) lipid for bilayer formation and included a fraction of 1,2-dioleoyl-*sn*-glycero-3-phospho-L-serine (PS) and/or L-α-phosphatidylinositol-4,5-bisphosphate (PIP2) to introduce negative charges on the bilayers. PS has one net negative charge at physiological pH while PIP2 has three. As shown in Figure 1A, bilayer formation was confirmed by the turning point in the plot of frequency vs. time and the negligible dissipation change (Jonsson et al., 2008). The final frequency shift is a few Hz more than expected from a pure DOPC bilayer, in agreement with the extra mass from the PIP2 lipids.

Binding of IRSp53 was verified qualitatively in many QCMD experiments (*n* > 20). An example is shown in Figure 1B to illustrate that the lipid composition had a negligible influence on protein binding as long as it contained a significant fraction (tens of percent) of negatively charged lipids. The lipid fractions represent those in the compositions used to prepare the vesicles and it is expected that the molar fractions are the same in the bilayer formed. In subsequent experiments, the main lipid composition used was 30% PS (mainly because it is cheaper than PIP2). The interaction was found to be irreversible, i.e., IRSp53 did not dissociate from the surface upon rinsing. In other words, the dissociation rate constant is zero and the affinity approaches infinity. Higher concentrations and longer binding times suggested a saturation signal of ~50 Hz, which in theory would be reached also for lower concentrations if waiting long enough (and wasting more protein by pumping new solution). At higher ionic strength (250 mM) the affinity became weaker and the interaction was reversible, i.e., the protein was released upon rinsing (not shown). This dependence on ionic strength confirms the electrostatic nature of the binding, which has been observed in many studies and even in simulations (Levtsova et al., 2011). In this study we want to elucidate the behavior of IRSp53 at physiological ionic strength (~150 mM), which was used in the following experiments.

**Figure 2:**
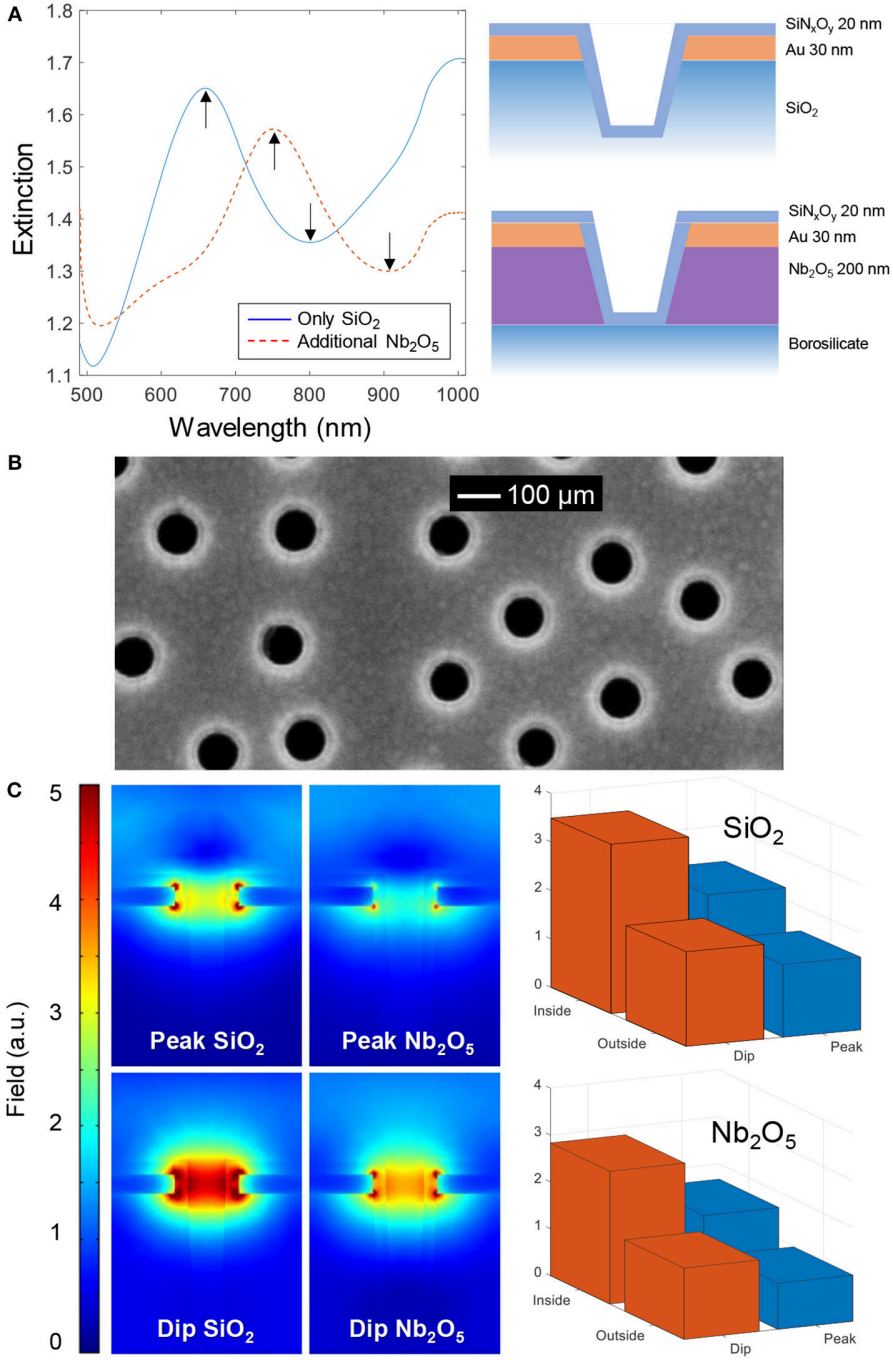
The two types of nanowells. **(A)** Extinction spectra in water and nanostructure schematics. **(B)** Electron microscopy image of nanowells in SiO_2_ coated with SiN_x_O_y_. **(C)** Simulated near fields at the peak and dip wavelengths for square arrays with periodicity of 300 nm in water. The bar plots show the average field strength on the planar surface (outside) vs. the curved surface exposed inside the nanowells (inside), in both cases 5 nm away from the SiN_x_O_y_ coating (which has refractive index 1.5).

To investigate the influence of membrane curvature, plasmonic nanowells were prepared as described in previous reports (Junesch et al., 2012; Malekian et al., 2017). We used two different types of nanowells consisting of niobia (Junesch et al., 2015) or silica (Ferhan et al., 2018) underneath the 30 nm gold film (Figure 2A). Although both these structures have been presented previously, we have not yet compared their sensing performance. Throughout the rest of this paper we refer to these as Nb_2_O_5_ and SiO_2_ nanowells. The aperture diameter in gold was tuned to ~100 nm by shrinking the colloids with oxygen plasma treatment (Xiong et al., 2016). Characteristic extinction spectra of the nanowells are shown in Figure 2A, in agreement with previous results (Junesch et al., 2015; Ferhan et al., 2018). The structures are identical in terms of the dimensions of the apertures and the only difference is that Nb_2_O_5_ has a higher refractive index (2.2) than SiO_2_ (1.5). This makes the asymmetric plasmon resonance (peak and dip) from the short-range ordered array of nanowells appear at longer wavelength for the case of Nb_2_O_5_ nanowells. Since the full spectrum is obtained in parallel from the spectroscopy setup, it is possible to track both the peak and the dip positions simultaneously with high temporal resolution (Junesch et al., 2015; Ferhan et al., 2018).

In all experiments, a ~20 nm conformal coating of silica or silicon nitride was first deposited by plasma enhanced chemical vapor deposition onto the nanowells. This insulating coating could not be fully visualized by electron microscopy but a reduction in nanowell diameter was observed after deposition when imaging from above (Figure 2B). For more images of nanowells and conformal coatings for bilayer formation we refer to previous studies (Junesch et al., 2012; Malekian et al., 2017; Ferhan et al., 2018), also from other groups (Im et al., 2010). Prior to membrane formation by vesicle rupture, samples were cleaned with oxygen plasma and thus we expected the silicon nitride coating to also contain a significant fraction of oxygen. Throughout this study no differences were observed among the coatings with respect to success of bilayer formation (see further below) and we refer to both coatings as SiN_x_O_y_ since their stoichiometry is unknown.

Our sensor concept for detection of binding to negative curvature, i.e., the tendency to bind preferentially inside the nanowells, is based on analyzing the relative shifts in the peak and dip wavelengths obtained from the extinction spectrum. Although these resonance features originate from the same optical phenomenon, they still behave differently in refractometric sensing experiments. In brief, the peak position corresponds to grating-type excitation of propagating modes by the short-range ordering of the nanowells, while the dip behaves as a localized resonance (Xiong et al., 2016). We have previously introduced a dimensionless parameter ζ to quantify the degree of affinity to negative membrane curvature (Junesch et al., 2015):

$$\begin{array}{l} \zeta =1-\frac{\Delta \lambda_{\text{peak}}}{\Delta \lambda_{\text{dip}}} \end{array}$$

Since the sensitivity (the spectral shift) to refractive index changes is always higher for the dip compared to the peak (Junesch et al., 2015; Xiong et al., 2016; Ferhan et al., 2018), ζ is always larger than zero (for instance, for bulk changes the dip shifts approximately twice as much as the peak). To bind to the planar surface, Δλ_peak_ can be almost as high as Δλ_dip_, which means that ζ is close to zero, while for binding inside nanowells, Δλ_peak_ can be close to zero so that ζ approaches one (Junesch et al., 2015).

Figure 2C shows the simulated near field at the peak and dip wavelengths for nanowells containing Nb_2_O_5_ or SiO_2_ as support. The simulated peak and dip wavelengths (680 and 720 nm for SiO_2_, 820 and 860 nm for Nb_2_O_5_) were found by calculating the far field spectra. These values are in fair agreement with the experimental peak and dip positions in Figure 2A (The experimental Nb_2_O_5_ nanowells were prepared with colloids that gave a shorter center to center distance of ~230 nm compared to the simulated period of 300 nm, which explains the relatively large difference between simulation and experiment in that particular case). The near field plots provide information about which regions of the nanostructure are most sensitive to refractive index changes (at a given wavelength). First, it is clear that the Nb_2_O_5_ layer, due to its higher refractive index, makes the field stronger on the lower side of the gold film, although the average field strength is higher for SiO_2_ nanowells. Further, we averaged the field strength ~5 nm from the solid surface inside the nanowell and compared it with the top planar surface, for both structures and for both resonance wavelengths (Figure 2C). These results confirm that, although the field is stronger inside the nanowells at both the peak and the dip wavelengths, the *ratio* of the dip and peak field strengths is higher upon binding to the interior of the nanowell compared to the planar surface region. This illustrates the location-specific detection concept and the utilization of the ζ parameter in a qualitative sense. However, the near-field plots are highly sensitive to the exact geometry of the nanostructure, which is hard to determine experimentally. Because of this we believe that there is a risk for overinterpreting this kind of simulation results if going into a too detailed quantitative analysis.

**Figure 3:**
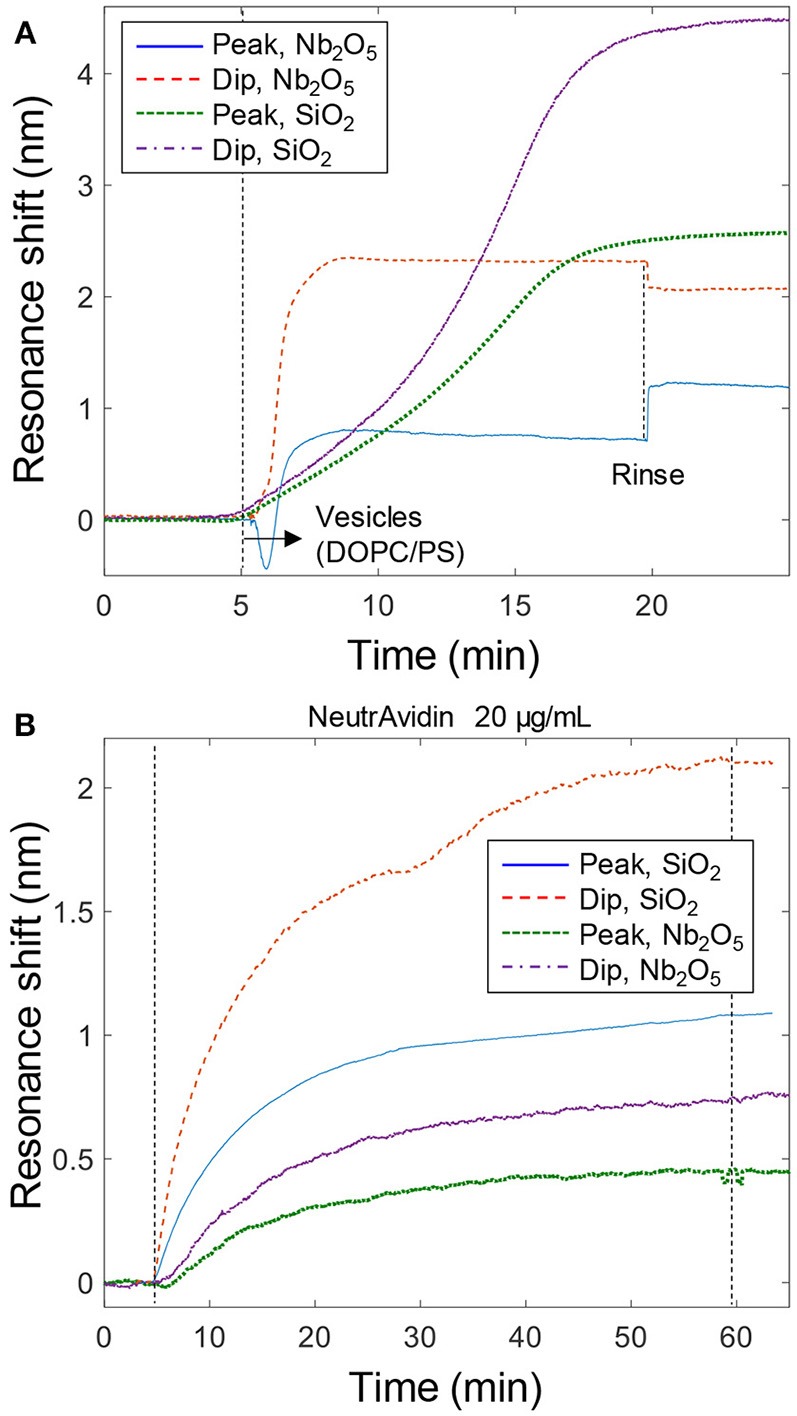
Bilayer formation and model protein binding. **(A)** Signals from bilayer formation (with vesicles containing PS) on the nanoplasmonic sensor surfaces. An ~5 times higher concentration of vesicles (~300 μg/mL) were introduced to the Nb_2_O_5_ structures, which is why a step-like change is observed upon liquid exchange (This is due to light scattering by vesicles in the bulk). **(B)** Example data of binding of NeutrAvidin to bilayers with biotinylated lipids. Peak and dip signals are shown for both structures.

Bilayer formation on the different nanowells with vesicles containing negative PS lipids was monitored in real-time (Figure 3A). For SiO_2_ nanowells, the kinetics showed the characteristic acceleration (second derivative becomes zero) which confirms vesicle rupture (Jonsson et al., 2007, 2008; Ferhan et al., 2018). For Nb_2_O_5_ nanowells, this effect is less clear in the data, which is most likely related to the differences in near field distribution (Figure 2C). Still, it is known that bilayer formation is possible (Junesch et al., 2015), as expected since the SiN_x_O_y_ coating and the dimensions are the same for both types of nanowells. To ensure that bilayer formation did occur on Nb_2_O_5_ nanowells before introducing proteins, we confirmed that the kinetics and the dip/peak ratio were different when compared to vesicle adsorption without rupture (which is what occurs on nanowells without the SiN_x_O_y_ coating). In brief, if there is no bilayer formation, the dip/peak signal ratio is lower (lower ζ) as intact and immobile vesicles are too large to enter the nanowell interior. Also, the kinetics are mass transport limited to a higher extent, so that the signal does not saturate as abruptly as in Figure 3A. Furthermore, fluorescence recovery after photobleaching has already been used to confirm fluid bilayers on both the Nb_2_O_5_ (Junesch et al., 2015) and the SiO_2_ (Ferhan et al., 2018) nanowells. Oxygen plasma and/or surfactants were used to clean and reuse sensor surfaces at least a few times, but eventually the coating was no longer able to support membrane formation from vesicles (in this case a new coating can be applied). In all experiments used in the analysis below, we first confirmed bilayer formation from the mentioned characteristic traits in the vesicle adsorption kinetics.

In order to establish the expected value of ζ for a protein binding to the lipid bilayer without any curvature preference, we introduced NeutrAvidin to bilayers containing a small fraction of biotinylated lipids (Junesch et al., 2015). Example data is shown in Figure 3B, yielding a value close to ζ = 0.4 for both SiO_2_ and Nb_2_O_5_ nanowells. Repeated measurements (*n* = 4) gave an uncertainty of 0.05 for the same structure and the variation barely increased when including data from both types of nanowells (it remained below 0.1). This is in agreement with previous results on similar Nb_2_O_5_ nanowells which showed ζ = 0.5 ± 0.1 for NeutrAvidin (Junesch et al., 2015). In fact, the ζ values for NeutrAvidin binding were the same (within the error) as for the completed bilayer formation (ζ = 0.45 ± 0.1, *n* = 10). This is expected since the protein, which is a couple of nm in size, accumulates just on top of the ~5 nm thick bilayer, i.e., the lipids and the protein are localized at roughly the same positions. Hence it is also possible to use the peak and dip signals from bilayer formation to calculate a reference ζ value, which is then used to evaluate if the subsequent protein binding exhibits a preference for negative curvature (it is important that the final values of peak and dip signals for the complete bilayer are used though, see further below).

**Figure 4:**
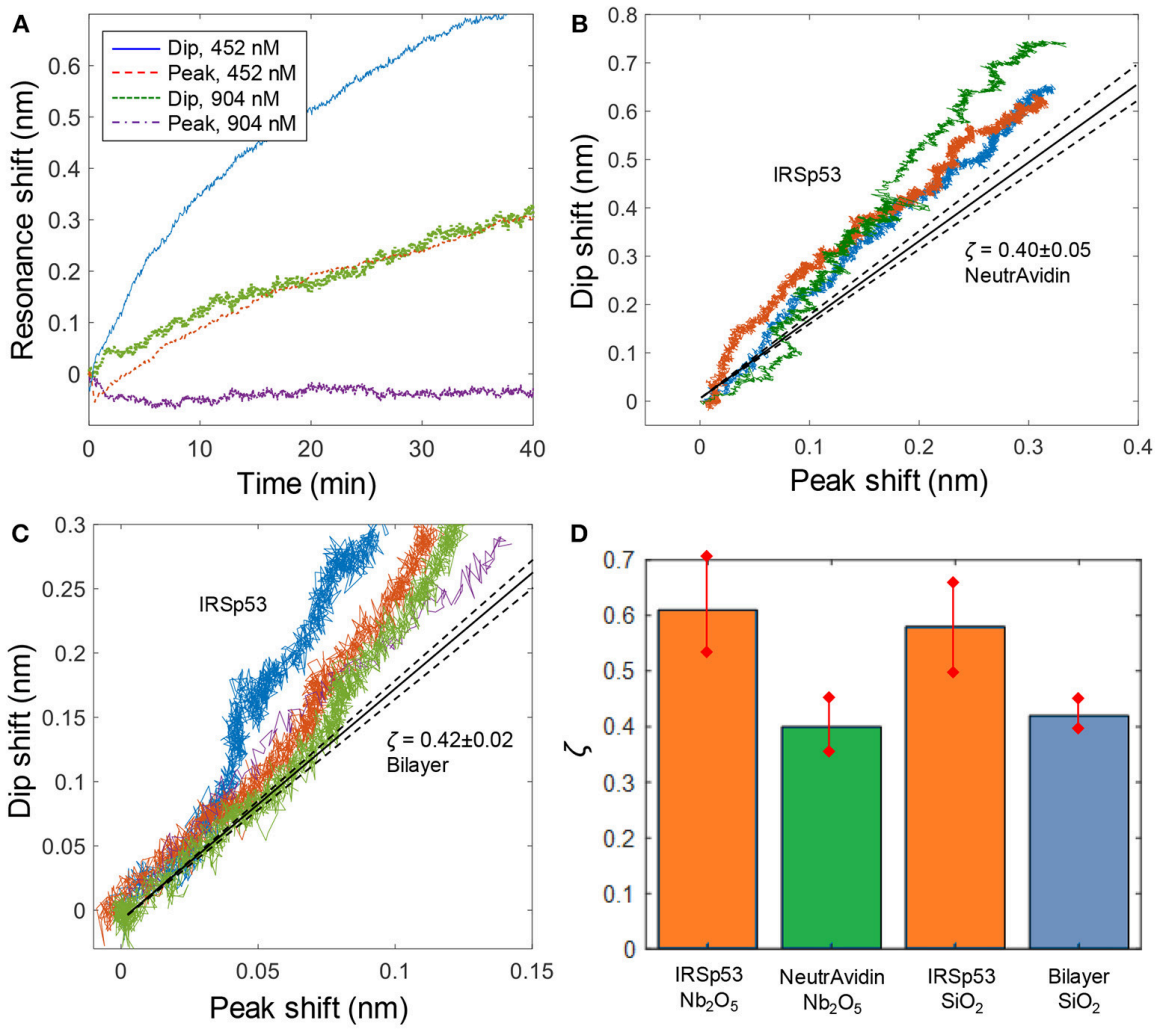
IRSp53 binding with negative curvature preference. **(A)** Kinetics of IRSp53 binding (introduced at 0 min) to membranes with negative lipids on Nb_2_O_5_ nanowells. For the lower concentration no peak shift is detected. **(B)** Dip and peak signals for IRSp53 compared to NeutrAvidin binding to Nb_2_O_5_ nanowells. **(C)** Dip and peak signals for IRSp53 compared to bilayer formation on SiO_2_ nanowells. **(D)** Summary of ζ values with error margins. For Nb_2_O_5_ nanowells, NeutrAvidin is used as the reference and for SiO_2_ nanowells, the preceding bilayer is used.

After verifying bilayer formation and binding of a protein with no preference with respect to surface curvature, we measured the plasmonic response (peak and dip) upon binding of IRSp53, starting with Nb_2_O_5_ nanowells. Figure 4A shows two examples of IRSp53 binding at different concentration. To visualize the curvature dependence, we plotted the binding process as dip vs. peak shifts for several IRSp53 experiments (Figure 4B). The shape of these plots provides information about curvature preference during the binding process. It should be kept in mind that the bilayer is a 2D liquid environment and that bound molecules can diffuse on the surface to find the geometry that they prefer. It is also important to note that, especially for the irreversible IRSp53 binding, no curvature preference will be detected when the whole surface is saturated because the protein also still binds to planar membranes (Figure 1B). When all nanowells are filled up, binding will continue and eventually a value of ζ will be reached which does not differ from that measured upon NeutrAvidin binding. Therefore, the dip vs. peak shift plots should not be linear, i.e., the dip should shift faster initially, and it is for low absolute signals before surface saturation that the curvature preference can be identified. This is in fair agreement with the plots in Figure 4B. As controls, for NeutrAvidin the plots were linear as expected (Junesch et al., 2015) and for bilayer formation the shape was inversed since adsorption of intact vesicles occurs primarily to the planar surface (not shown). The vesicles were extruded through 30 nm pores and are thus comparable to the nanowells in diameter.

To identify the curvature effect, we analyzed out-of-equilibrium data for IRSp53 where the surface has not yet become entirely covered, so that the protein has the possibility to accumulate inside nanowells. As an example, we analyzed data points when the peak shift had reached 0.2 nm, which is less than half of the saturated signal of ~0.5 nm peak shift for Nb_2_O_5_ nanowells. This gave a value of ζ = 0.61 ± 0.09 (based on *n* = 10 experiments), which is significantly higher than for NeutrAvidin. We also evaluated the ζ values obtained for IRSp53 binding to SiO_2_ nanowells (Figure 4C), resulting in ζ = 0.58±0.08 (*n* = 4) when the peak shift had reached 0.1 nm. Here the bilayer values were used as a reference, which gave ζ = 0.42 ± 0.03 (*n* = 8).

Overall, our results show that IRSp53 indeed has a preference to bind to negative membrane curvature, although the differences in ζ are less obvious in comparison with previous work of viral capsids that accumulate at negative curvature due to multivalent interactions (Junesch et al., 2015). A large number of experiments were summarized to obtain error bars and ensure statistical significance (Figure 4D). For SiO_2_ nanowells, the contrast in ζ is still not very high compared to the experimental uncertainty and it was necessary to look at data at low coverage of IRSp53, i.e., early in the binding process, to identify the difference in ζ. For Nb_2_O_5_ nanowells, very higher values of ζ could sometimes be obtained (e.g., ζ ≈ 1 for 452 nM in Figure 4A). Such high values were, however, not acquired in a reproducible manner. This is most likely related to small differences in dimensions and shape of the nanowells, which can have a major influence on the near field distribution. This kind of structural variation could originate from, for instance, poor reproducibility in the dry etching step.

## Conclusions

We have for the first time used plasmonic nanowells to confirm preferential binding of a protein to lipid membranes with negative curvature compared to regions with planar geometry. The main strength of this sensor is that it is relatively simple and provides real-time analysis of binding in a label-free format. The main limitation is the inability to detect if proteins *induce* curvature since the membrane simply follows the surface topology. It is believed that curvature is induced by IRSp53 at a certain surface concentration (Callan-Jones and Bassereau, 2013). Thus, our sensor can only in part verify the proposed mechanisms and many functions of a protein such as IRSp53 (Kang et al., 2016). Nevertheless, we consider it an important step to verify the curvature binding preference with an independent method. It should also be noted that the sensor is only semi-quantitative with respect to the accumulation at negative membrane curvature. A higher value of ζ (in comparison with the bilayer or NeutrAvidin) would certainly suggest a stronger effect, but it is hard to quantify exactly how many proteins are inside the nanowells. Further, the nanowells have a range of curvatures along the vertical axis due to their shape. IRSp53 should prefer ~0.05 nm^−1^ (Prévost et al., 2015), which is within the expected curvature range inside the nanowells (top diameter ~80 nm). It seems likely that if one could prepare deep nanowells with perfectly vertical walls and the right diameter, the preferential binding inside nanowells would be even more pronounced (higher ζ).

We believe that our sensor will be a valuable complementary tool for investigating processes leading to the generation of highly curved membranes, such as virus budding, cell division or filopodia formation, to name a few examples. One can also imagine connections to naturally curved membranes found in organelles such as the Golgi apparatus and the mitochondrion. In the future, other types of nanostructures with more clearly defined geometry could be investigated for the same purpose, such as cylindrical channels in hollow nanowires (Sköld et al., 2010). However, the plasmonic nanowells are quite unique since they provide a built-in optical label-free sensor and a spectral signature for binding at negative curvature.

## Author Contributions

All authors contributed with experimental work and/or analysis and interpretation. Experiments were performed mainly by GE and ER. The project was initiated by AD and MB. BM and KX fabricated nanostructures. JM and F-CT prepared and purified the protein. N-JC contributed with supplemental data. AD wrote the manuscript with input from the coauthors.

### Conflict of Interest Statement

The authors declare that the research was conducted in the absence of any commercial or financial relationships that could be construed as a potential conflict of interest.
